# Breast cancer screening practice and its associated factors among women in Kersa District, Eastern Ethiopia

**DOI:** 10.11604/pamj.2019.33.144.18062

**Published:** 2019-06-26

**Authors:** Teshale Mulatu Dibisa, Tilayie Feto Gelano, Lemma Negesa, Tewelde Gebre Hawareya, Degu Abate

**Affiliations:** 1 Department of Midwifery, School of Nursing and Midwifery, College of Health and Medical Sciences, Haramaya University, Harar, Ethiopia; 2 Department of Pediatrics and Neonatal Nursing, School of Nursing and Midwifery, College of Health and Medical Sciences, Haramaya University, Harar, Ethiopia; 3 Department of Clinical Nursing, School of Nursing and Midwifery, College of Health and Medical Sciences, Haramaya University, Harar, Ethiopia; 4 School of Medical Laboratory, Department of Microbiology, College of Health and Medical Sciences, Haramaya University, Harar, Ethiopia

**Keywords:** Breast cancer, breast screening practice, Eastern Ethiopia

## Abstract

**Introduction:**

Breast cancer is one of the most common cancers and cause of death among women globally. Mortality due to breast cancer was higher in lower (LMICs) and middle-income countries than high income countries (HICs) mostly due to lack of timely detection and treatment. There was limited evidence related to breast cancer screening practice among women in Eastern Ethiopia. Therefore, the aim of this study was to assess breast cancer screening practice and its associated factors among women in this area.

**Methods:**

A community based descriptive cross-sectional study design was conducted among 422 randomly selected women in Kersa district, Eastern Ethiopia using systematic sampling. Data were collected using pretested interviewer administered questionnaire. Logistic regression was used to analyse the association between the dependent and independent variables.

**Results:**

The overall breast cancer screening practice among women was 6.9%. Women with the age of 26 years and above were 2.3 times more likely to have breast cancer screening practice as compared to women with age of 20-25 years (AOR=2.3; 95% CI: 1.4, 3.7), and women who had good knowledge on breast cancer risk factors were 3.4 times more likely to had breast cancer screening as compared to their counterpart (AOR=3.4; 95% CI: 1.3, 9.4). The women who had ever heard about breast cancer screening were 2.8 times more likely to have breast cancer screening as compared to those who had never heard about breast cancer screening (AOR=2.8; 95% CI: 1.2, 6.5).

**Conclusion:**

The overall breast cancer screening practice was very low among women in the study area. Age and women's knowledge towards breast cancer risk factors and breast cancer screening information were identified as important factors for breast cancer screening practice.

## Introduction

Breast cancer is a malignant tumor which starts in the cells of the breast and grows into or metastasize to surrounding or distant areas of the body [1]. It is one of the most common cancers and cause of death among women globally [2-4]. In 2012, about 14.1 million women were diagnosed with cancer, of which 1.7 million were breast cancer cases; 56.8% of the cases were from low-income countries. Some 522 000 deaths due to breast cancer were recorded the same year, with the majority from sub-Saharan Africa (SSA). When compared with the WHO report of 2008, the incidence of breast cancer is increasing with high pace and it is expected to reach over 19.3 million among women by 2025, with the majority from sub-Saharan African (SSA) [5]. Over the past two decades, breast cancer has become a matter of serious public health concern in developing countries due to a high pace increase of its incidence. This burden of breast cancer is increasing because of different factors like aging, smoking, obesity, physical inactivity, and other poor health related behaviors [6]. Mortality due to breast cancer was higher in LMICs than HICs mostly due to lack of timely detection and treatment. This problem was more aggravated by rising burden of breast cancer incidence [7]. In sub-Saharan Africa including Ethiopia, cancer is one of non-communicable diseases, which is causing illnesses and leading death [8,9]. It was estimated that around 10,000 Ethiopian women had breast cancer with thousands of more cases unreported [10]. Only about 500 patients (less than 1%) per year got treatment services. In addition, the treatment may cost more than 80,000 ETB (8,335 USD) which most patients cannot afford [11].

Moreover, most healthcare facilities in Ethiopia do not have advanced laboratory investigations for diagnostic breast cancer screening (BCAS) because of resource scarcity in the country [12,13]. Timely detection of breast cancer is strongly recommended because of better treatment prognosis with more effective cost [14]. Evidence also showed that diagnosis delay of three to six months was associated with advanced stage breast cancer and lower survival rate [15]. Breast cancer detection require awareness of breast cancer risk factors, signs and symptoms using breast screening methods such as breast self-examination (BSE), clinical breast examination (CBE) and diagnostic assessment like mammography [16]. Regardless of the above facts, very little was known about breast cancer screening practice among women and its associated factors in Eastern Ethiopia. Therefore, the aim of this study was to assess breast cancer screening practice and its associated factors among women in Eastern Ethiopia.

## Methods

**Study area and period:** the study was conducted from 1^st^ to the 30^th^ May 2017 in Kersa district, Eastern Hararghe zone of Oromia regional state, Eastern Ethiopia. Kersa is one of the 180 districts in the Oromia region. It is located between 41040”0’ and 41057”30’ (longitude) and 09015”15’ and 09029”15’ (latitude) [17]. There are 35 rural sub-districts (called Kebeles) and 3 small towns. According to the 2007 national census, the district has a total population of 172,626 of whom 6.9% are urban dwellers, and a population density of 372 people per square kilometer. The sex ratio and number of persons per household were 1.0 and 5.1 respectively. The annual net population growth is 1.6. In different years, the total fertility rate ranges from 4.0 to 5.3. The district capital is Kersa, which is 44km far from Harar to the west [17,18].

**Study designs:** a descriptive community based cross-sectional study was implemented.

**Source population:** all women who were living in Kersa district during the study period.

**Study population:** all women who were living in selected kebeles of Kersa district during the study period.

**Inclusion and exclusion criteria:** inclusion; all women who were living in selected Kebeles of the study area during the study period. Exclusion; all women who were seriously ill, had mental derangement, or had hearing problems were excluded from this study.

**Sample size estimation:** single population proportion formula was used with assumption of 5% margin of error and 95% confidence level. Assuming 50% of population proportion with the practice of breast cancer screening (no previous similar study) and adding 10% of non-response rate the total estimated sample size was 422 women.

$$n=\frac{z^{2}P(1-P)}{d^{2}}$$

n= (1.96)²(0.5x0.5)/(0.05)²= 384.2 = 384 + 10% non-response rate = 384 + 38.4 = 422.4 ~ 422

**Figure 1:**
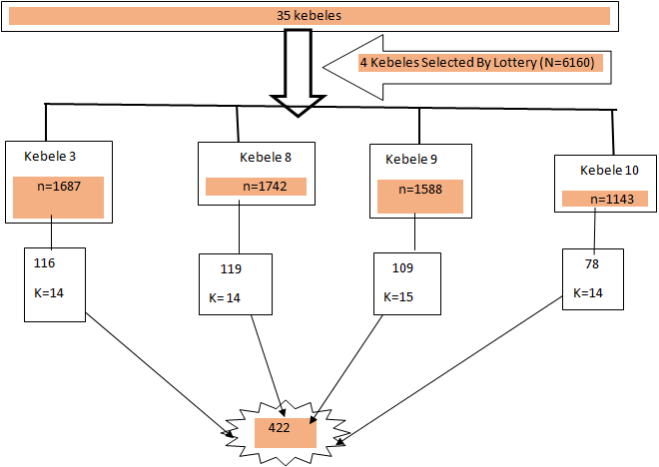
Schematic diagram of sampling technique

**Sampling technique:** initially, from a total of thirty five kebeles four kebeles were selected by lottery method. Then study subjects were allocated to selected kebeles proportionally to their size. Total number of women in selected kebeles were obtained from Kersa demographic health surveillance (KDHS). The households were selected through systematic sampling. Finally, the study subjects were drawn from the selected households (Figure 1).

**Data collection procedure:** the data collection tools were first prepared in English and then translated into local language for data collection purpose. One day training was provided to the data collectors and supervisors on objectives, data collection tools and procedures by the principal investigator. Three diploma holder midwives carried out data collection. The questionnaires were pre-tested on 5% of total sample size at site different from actual study area to ensure its validity. The collected data was checked for accuracy and completeness on daily basis and supervised by two BSc midwives. Written informed voluntary consent was obtained from each participant prior to data collection. After data collection, it was retranslated back to English language for analysis and maintain internal consistence.

**Data processing and analysis:** collected data were checked for completeness, coded and entered into EPI-Info-7 by using double data entry method. SPSS version-20 was used for analysis. Data cleaning was done to check forgotten entries, consistency and outliers. Frequencies of variables were generated; tabulation and percentages were used to illustrate the study findings. Hosmer-Lemeshow's and Omnibus tests was done to test for model fitness. Bivariate logistic regression was used to identify factors associated with breast cancer screening practice. All variables with p-value ≤ 0.2 were taken into the multivariable model to control for all possible confounders. Finally, the results of multivariable logistic regression analysis were presented in crude and adjusted odds ratio with 95% confidence intervals. Level of statistical significance was declared at p-value less than 0.05.

### Measurements

**Knowledge of women on breast cancer risk factors:** this part was assessed by using reference guidelines of the American Cancer Society [1]. There were sixteen questions related to breast cancer risk factors. For each question with three options (Yes, No and Do Not Know). Responses of “Yes” were recorded as 1 whereas “No” and “Do Not Know” were scored as 0. Total knowledge score above the mean was considered as good knowledge whereas below the mean score was considered as poor knowledge.

**Knowledge of women on breast cancer signs and symptoms:** ten questions related to breast cancer signs and symptoms were used with three options (Yes, No and Do not know) and responses of “Yes” were recorded as 1 whereas “No” and “Do Not Know” were scored as 0. Total of knowledge score on signs and symptoms of breast cancer above the mean was considered as *good knowledge* whereas below the mean score was considered as *poor knowledge*.

**Breast cancer screening practice:** if the mother had ever practiced breast cancer screening methods

**Knowledge of breast cancer screening methods:** mother is considered knowledgeable if she knows at least one screening method.

## Results

**Socio-demographic characteristics of study participants:** 422 women were interviewed. Out of this 422, about one third of the study participants (33.4%) were between the age of 20-25 years and the mean age of study participants was 32.6 years with 9.5 standard deviation. The minimum age was 20 while the maximum age was 65. Regarding to educational status, majority of study participants (94.5%) were illiterate. Most of the study participants (85.5%) were married. Concerning ethnic group and religion, majority of the participants (96.2%) were Oromos and Muslims (96.7%) respectively (Table 1).

**Table 1:** Socio-demographic characteristic of study participants in Kersa District, Eastern Ethiopia, 2017 (n = 422)

Variables	Categories	Frequency	Percent (%)
Age in years	20-25 years	141	33.4
	26 years and above	281	66.6
Educational status	Illiterate	399	94.5
	Literate	23	5.5
Marital status	Single	21	5.0
	Married	361	85.5
	Divorced &widowed	40	9.5
Occupation	Housewife	413	97.9
	Others*	9	2.1
Ethnicity	Oromo	406	96.2
	Amhara	11	2.6
	Harari	5	1.2
Religion	Muslim	408	96.7
	Orthodox	8	1.9
	Protestant	6	1.4
Monthly income	120-2000EB	272	64.5
	2001-3500 EB	123	29.1
	>=3501 EB	27	6.4

* unemployed, self-employed and merchants, EB: Ethiopian Birr

**Breast cancer screening practice of the women:** the study revealed that only 6.9% women had ever practice of BCAS either BSE (3.6%) or CBE (5.5%) respectively and no study participants had undergone mammography (Table 2).

**Table 2:** Breast screening practices among women in Kersa District, Eastern Ethiopia, 2017 (n = 422)

Variables	Categories	Frequency	Percent (%)
Ever had breast cancer screening	No	393	93.1
	Yes	29	6.9
Breast self-examination	Yes	15	3.6
	No	407	96.4
Clinical breast examination	Yes	23	5.5
	No	399	94.5
Mammogram	Yes	0	0
	No	422	100

BCA: breast cancer, BCAS: breast cancer screening

**Knowledge of women towards breast cancer risk factors, signs and symptom:** this study showed that more than half of women (51.2%) had good knowledge about breast cancer signs and symptoms, but only 5.5% of study subjects had good knowledge about breast cancer risk factors. Out of the total, majority (90.8%) of women stated that early menarche is risk factor for breast cancer followed by due to late menopause (74.9%) and family inheritance (73.9%). Only few women stated cigarette smoking (2.8 %) and alcohol consumption (1.4%) as risk factor for breast cancer respectively (Table 3). Concerning women's knowledge about signs and symptoms of breast cancer majority (87.9%) of study participants stated that pain or soreness in the breast is symptom of breast cancer followed by ulceration of the breast (87.7%), inversion of nipple (87.7%) and discharge from the breast (81.8%) respectively (Table 4).

**Table 3:** Knowledge of women towards breast cancer risk factors in Kersa District, Eastern Ethiopia, 2017 (n = 422)

Variables	Frequency	Percentage %
**Results due to lack of breast feeding**	276	65.4
**Smoking increases its risk**	12	2.8
**Alcohol consumption**	6	1.4
**may be inherited**	312	73.9
**Personal history of breast cancer**	51	12.1
**may result from obesity**	9	2.1
**high fat diets**	13	3.1
**Early menarche (< 12 years**	383	90.8
**Late menopause (> 55 years)**	316	74.9
**Deficiency of physical activity**	114	27.0
**Environmental pollution**	267	63.3
**Aging**	218	51.7
**Late age at first full-term pregnancy (> 30 years)**	165	39.1
**Recent oral contraceptive use**	18	4.3
**Recent and long-term use of hormone replacement therapy**	128	30.3
**High-dose radiation to chest**	8	1.9

**Table 4:** Knowledge of women about signs and symptoms of breast cancer in Kersa District, Eastern Ethiopia, 2017 (n= 422)

Variables	Frequency	Percent (%)
**Lump in the breast**	198	46.9
**Discharge from the beast**	345	81.8
**Pain or soreness in the breast**	371	87.9
**Change in the size of the breast**	217	51.4
**Discoloration /dimpling of the breast**	279	66.1
**Ulceration of the breast**	370	87.7
**Changes in the shape of the breast**	261	62
**Inversion/pulling in of nipple**	370	87.7
**Swelling or enlargement of the breast**	178	42.2
**Scaling/dry skin in nipple region**	144	34.1

**Knowledge of women about breast cancer screening:** overall, the study finding indicated that only 6.6% of study participants ever heard about breast cancer screening and 5.2% of them knew about breast cancer screening methods. With regards to types of examination or screening, about 5% of women knew breast self-examination, and 4.5% of the women knew clinical breast examination and no study subjects knew about mammography screening (Table 5).

**Table 5:** Women’s knowledge on breast cancer screening in Kersa District, Eastern Ethiopia, 2017 (n= 422)

Variables	Category	Frequency	Percent (%)
Ever heard about BCAS	No	394	93.4
	Yes	28	6.6
Know breast screening methods	No	400	94.8
	Yes	22	5.2
Women know CBE	No	403	95.5
	Yes	19	4.5
Women know BSE	No	401	95
	Yes	21	4.97
Know mammography	No	422	100
	Yes	0	0

BCAS: breast cancer screening, BSE: breast self-examination, CBE: clinical breast examination

**Breast cancer screening practice and its associated factors:** after adjusting for other variables, only three variables remained significant in multivariate logistic regression. Women with the age of 26 years and above were 2.3 times more likely to have breast cancer screening practices as compared to women with age category of 20-25 years (AOR=2.3;95% CI:1.4,3.7). The study participants who had ever heard about breast cancer screening were 2.8 times more likely to have breast cancer screening compared to women who had never heard about breast cancer screening (AOR=2.8; 95% CI: 1.2, 6.5). Women who had good knowledge about breast cancer risk factors were 3.4 times more likely to have breast cancer screening compared to their counterpart (AOR=3.4;95% CI: 1.3, 9.4) (Table 6).

**Table 6:** Bivariate and multivariate analysis of factors associated with breast cancer screening practice among women in Kersa Districts, Eastern Ethiopia, 2017 (n= 422)

Variable	BCA screening practice		Odds ratio (95% CI)	
	Poor	Good	Crude	Adjusted
**Age in years**				
20-25years	104	37	1	1
>+26years	168	113	1.89 (1.2, 2.9)	**2.3 (1.4, 3.7)**
**Educational**				
Illiterate	261	138	1	1
Literate	8	15	3.55 (1.47, 8.57)	2.5(0.89, 6.5)
**Ever heard about breast CA**				
No	179	88	1	1
Yes	89	66	1.51(1, 2.3)	1.2 (0.75, 1.8)
**Ever heard about BCA screening**				
No	258	136	1	1
Yes	9	19	4 (1.8, 9.1)	**2.8 (1.2, 6.5)**
**Knowledge about BCA**				
Poor knowledge	145	63	1	1
Good knowledge	123	91	1.7(1.14, 2.54)	2.5 (0.6, 10.3)
**Knowledge on S/S of BCA**				
Poor knowledge	142	64	1	1
Good knowledge	126	90	1.585(1, 2.4)	0.6 (0.14, 2.3)
**Knowledge on BCA risk factors**				
Poor knowledge	261	138	1	1
Good knowledge	7	16	4.3(1.7, 10.8)	**3.4 (1.3, 9.4)**

BCA: breast cancer, S/S: signs and symptoms

## Discussion

The finding from current study showed that overall breast cancer screening practice among women was 6.9%. This finding was very lower than the study from Benin (63.5%) and India (46.6%) [16,19]. This might be due to lack of community base awareness and lack of breast cancer screening program in our country, specifically in the study area. Out of the total study participants, 5.5% performed CBE and only 3.6% of the women performed BSE. This is consistent with the study done in north Ethiopia which reported only 26 (6.5%) of study participants ever practice breast self-examination and only 25 (6.25%) of them practice breast self-examination regularly [20]. The study is also comparable with the study done in Iran which showed that only 10.1% of study subjects have performed BSE regularly as once per month and only 8.4% had CBE regularly as once per year and in Egypt only 1.3% practice BSE regularly every month and 6.1% reported that they performed it irregularly [21,22]. The finding is lower than other study in Ethiopia which reported poor practice of BSE (35.5%), CBE (32.5%) and mammography (16%) and that of Pakistan were 58 (19%) of women reported to have undergone clinical examination of breast for some breast complaint and 42 (13.8%) were investigated at some time [23,24]. The difference may be due to difference in educational status, study area and accessibility to information and composition of the study population.

This study showed that more than half of women (51.2%) had good knowledge about breast cancer signs and symptoms. This finding was lower than other findings from Ethiopia which reported high level of knowledge towards breast cancer among study participants [25]. This variation might be related to study subject differences as the previous study was conducted among nurses who may have more information or awareness about breast cancer. This study showed that only 6.6% of study participants ever heard about breast cancer screening of which 5.2% of the respondents knew about breast cancer screening methods. With regard to types of examination or screening, about 2.8% of women know about breast self-examination, and 4.5% of the women know about clinical breast examination, but no study subjects know about mammography. The finding is lower than that of Iran which showed that 21% and 9% have respectively heard about breast clinical examination and mammography [26]. This study finding is inconsistent with that of Saudi Arabia which showed that BSE was the most familiar method (43.4 %), clinical breast examinations (CBE) came next (28.2%) and mammography was the least identified method (9.3%) [27]. The difference may be due to variation of socio-demographic and socio-economic characteristics of the study participants as this study was conducted in rural area.

The current study also showed that only 5.5% of study participants had good knowledge about breast cancer risk factors. This result is incomparable with the studies done in Egypt (73%), Addis Ababa, Ethiopia (85%) and Nigeria (63%). The main reasons of this discrepancy could be related to level of awareness of the participants [22,23,28]. This study also attempted to identify factors, which are associated with breast cancer screening practice among women. The study showed that, women with the age of 26 years and above were 2.3 times more likely (AOR=2.3, 95% 1.4, 3.7) to have breast cancer screening practice as compared to women of 25 years age or less. This finding was in line with studies conducted in Australia and Nigeria [15,28]. This might be related to perception that as people get older, their risk of getting cancer increases. When women get older, their likely hood to have breast cancer screening practice increases.

The study also showed that women who had ever heard about breast cancer screening, were 2.8 times more likely to have breast cancer screening practices as compared to women who had not heard about it (AOR=2.8; 95%CI: 1.2, 6.5). Women who had good knowledge about breast cancer risk factors, were 3.4 times more likely to have breast cancer screening practice as compared to their counterparts (AOR=3.4;95% CI: 1.3, 9.4). This in line with the study conducted at India and Saudi Arabia which showed significant association between knowledge of women on breast cancer and breast cancer screening practice [19,29]. This is the fact that knowledge of women towards breast cancer can increase their understanding about the advantages of breast cancer screening.

**Limitation of the study:** the findings of this study were based on self-report, as it was not possible to validate claims made by respondents in the course of questionnaire administration.

## Conclusion

In conclusion, the overall breast cancer screening practices among women in the study area was very low. The factors such as women's age, knowledge towards breast cancer risk factors and information about breast cancer screening, were identified as important factors for breast cancer screening practice. Collaboration is needed between different sectors to increase community awareness towards breast cancer and endorse breast cancer screening policy to reduce morbidity and mortality related to breast cancer among women.

### What is known about this topic

- Breast cancer is the leading cause of mortality from all types of cancer occurring among women of reproductive age groups in Ethiopia followed by cervical cancer.

### What this study adds

- This study will enable the study participants to know breast cancer screening methods for early detection and prevention of breast cancer.

## Competing interests

The authors declare no competing interests.
